# A randomized, controlled trial of an innovative, multimedia instructional program for acquiring auditory skill in identifying pediatric heart murmurs

**DOI:** 10.3389/fped.2023.1283306

**Published:** 2024-01-16

**Authors:** Robin W. Doroshow, Julie Aldrich, Rebecca Dorner, Laurie Lyons, Robert McCarter

**Affiliations:** ^1^ Department of Cardiology, Children’s National Hospital and George Washington School of Medicine and Health Sciences, Washington, DC, United States; ^2^ Department of Pediatrics, Children’s National Hospital and George Washington School of Medicine and Health Sciences, Washington, DC, United States; ^3^ Department of Pediatrics, Georgetown University School of Medicine, Washington, DC, United States; ^4^ Department of Instructional Design and Technology, George Washington University School of Medicine and Health Sciences, Washington, DC, United States; ^5^ Division of Biostatistics, Children’s National Hospital and George Washington School of Medicine and Health Sciences, Washington, DC, United States

**Keywords:** cardiac auscultation, multimedia learning, pediatric murmurs, teaching auscultation, children's heart murmurs

## Abstract

**Objective:**

To create a brief, acceptable, innovative method for self-paced learning to enhance recognition of pediatric heart murmurs by medical students, and to demonstrate this method's effectiveness in a randomized, controlled trial.

**Materials and methods:**

A curriculum of six 10-min online learning modules was designed to enable deliberate practice of pediatric cardiac auscultation, using recordings of patients' heart murmurs. Principles of andragogy and multimedia learning were applied to optimize acquisition of this skill. A pretest and posttest, given 4 weeks apart, were created using additional recordings and administered to 87 3rd-year medical students during their pediatric clerkship. They were randomized to have access to the modules after the pretest or after the posttest, and asked to use at least the first 2 of the modules.

**Results:**

47 subjects comprised the Intervention group, and 40 subjects the Control group. On our primary outcome, distinguishing innocent from pathological with at least moderate confidence, the posttest scores were significantly higher for the Intervention group (60.5%) than for the Control group (20.0%). For our secondary outcomes, the 2 groups also differed significantly in the ability to distinguish innocent from pathological murmurs, and in identifying the actual diagnosis. On all 3 outcomes, those Intervention group subjects who accessed 4–6 modules scored higher than those who accessed 0–3 modules, who in turn scored higher than the Control group.

**Summary:**

Applying current principles of adult learning, we have created a teaching program for medical students to learn to recognize common pediatric murmurs. Its effectiveness was demonstrated in a randomized, controlled trial. The program results in a meaningful gain in this skill from 1 h of self-paced training with high acceptance to learners.

## Introduction

Congenital heart defects (CHD) occur in approximately 1% of live births, and can have serious effects on a child's health. Critical CHD can be detected with high sensitivity in the newborn using pulse oximetry (1). Most non-critical CHD present in childhood with an asymptomatic heart murmur, detected by a primary care provider (PCP). Innocent, or normal, heart murmurs occur far more frequently, with incidence ranging from 50%–90% (2). Therefore, the PCP must be able to distinguish between innocent and pathological murmurs, in order to appropriately refer patients with potential CHD without referring an excessive number of children with innocent murmurs (3–7). Hence, education in pediatric cardiac auscultation (CA) is routinely included in undergraduate and graduate medical education, and this skill has now been incorporated into standardized medical testing (8, 9).

Unfortunately, non-cardiologists have been repeatedly shown to be lacking in the skill of CA (10–16), suggesting that traditional methods of teaching it, such as lectures and bedside demonstration, are ill-suited to honing this sensory skill. Moreover, most non-cardiologists do not improve at CA after medical school (11, 17–20).

The recent evolution of digital technologies for recording and sharing audio signals has led to the development of new methods of teaching CA (12, 21) of the adult patient. While several such innovative programs have been successful in teaching subjects to identify an adult's cardiac diagnosis from a recording or simulation (22, 23), they cannot be applied to pediatric murmur education due to major differences in disease processes, heart rates, diagnostic prevalence, and presenting symptoms of CHD. Therefore, similar innovative programs are called for in pediatric CA (11, 13, 15–17, 24–28).

To enhance competency in this skill, we have produced a user-friendly online program for undergraduate medical students, using actual recordings from pediatric patients and applying central principles of andragogy (29) and multi-sensory learning (30). We have placed more emphasis on differentiating between innocent and pathological murmurs, which is the task of the pediatric PCP, than on arriving at a specific diagnosis (31, 32). We hypothesized that our method would improve acquisition of, and confidence in, this skill, compared with traditional approaches.

## Materials and methods

Six online modules, averaging 10 min each, were developed using multiple selected recordings from patients with confirmed cardiac diagnoses. The dominant principles of this teaching program are deliberate practice (14, 33), contiguity or linkage (34–36), and avoidance of cognitive overload (36–41).

Deliberate practice is a demonstrably effective method of improving performance; it is a form of practicing which is usually solitary, and requires considerable repetition and immediate feedback. Its most familiar application is in mastering a musical instrument. Contiguity refers to the learning advantage attained by placing closely related teaching points (e.g., auditory information coupled with visual information) near one another in time and/or space. Cognitive overload is a concept most simply represented as “too much information.” It is to be minimized in order to reduce extraneous processing of information, and has been well shown to interfere with acquisition of new skills.

The modules are accessed through the Internet with a computer or tablet, using standard earbuds. They are presented in a fixed sequence, starting with the Still's Murmur and Ventricular Septal Defects modules (Supplementary Table S1), allowing the learner to build upon each segment. To enable the application of deliberate practice, learners control the duration of playback of all recordings, and may review completed modules at will; and they participate in brief interactive activities scattered throughout the modules. Throughout the modules, they have free access to a set of recordings of 8 common murmurs and one of normal heart sounds for comparison (Supplementary Figures S1–S5).

To apply the principle of multimedia contiguity, we provided the learner with nonverbal content, such as the messages that (a) this starts with a simple binary decision, and (b) these children are normal and healthy. Additional relevant information depicted nonverbally includes (a) the common age of the child for that diagnosis, (b) the location of the stethoscope on the child's chest, (c) the side of the stethoscope used (bell or diaphragm), and (d) whether the child is upright or supine. This material is visually linked with each murmur heard, with the name of the diagnosis shown on the top of the screen, and with color-coding for each module. Limited, familiar voice narration (36) accompanies, but does not compete with, each murmur recording. Cognitive overload is minimized by targeting CA specifically, eliminating material targeting other goals of teaching in cardiology, such as knowledge of anatomy or pathophysiology.

We devised a 20-case pre-test, and a post-test containing the same 20 recordings in different order with pictures of another 20 child models. None of the test recordings was used in the teaching modules, which incorporated 115 different recordings. For each case, the learner was able to listen to the murmur as long as desired, and was then asked (a) Is this murmur innocent or not? (b) How confident are you in your answer? and (c) What do you think the cardiologist finds? (actual diagnosis). Demographic questions were included in the pre-test, and feedback questions in the post-test.

In a pilot study, these tests were administered preliminarily to 23 medical trainees and faculty in an iterative manner, to establish content validity and to provide a frame of reference for student performance. Recordings that were rarely identified correctly were eliminated, as were those felt by cardiologists not to be representative of the diagnosis.

The teaching program was offered to all 3rd-year medical students at George Washington School of Medicine and Health Sciences (GWU) over a 10-month period, during their required two-month pediatric clerkship. None had received previous instruction in pediatric auscultation. Students were randomized equally to a Control group and an Intervention group. All subjects took an online pre-test in person during a specified educational forum at the beginning of the clerkship, and a post-test similarly after 4 weeks. The Intervention subjects had access to the program between the two tests, and were asked to complete at least the first 2 modules. The Control subjects were given access after completion of the post-test. Entry into each module was automatically recorded. During the clerkship, all students had formal teaching and bedside instruction, and unlimited access to teaching programs available on the Internet (see Supplementary Table S2). All subjects received email-reminders during the interim to complete the modules or to individually learn about murmurs, depending upon group assignment.

Our primary outcome of interest was improvement in correct discrimination between innocent and pathological murmurs *with at least moderate confidence* (CDIP), as a surrogate for the decision of whether to refer the case for specialty evaluation. Confidence was rated on a 4-point Likert scale, 4 indicating “very confident.” Our secondary outcomes were improvement (a) in the simple distinction of innocent vs. pathological (DIP), and (b) in the correct identification of specific diagnoses. Total confidence scores were the sum of the 20 confidence responses.

To maintain confidentiality, all students were assigned study numbers. Results could therefore not affect grades for the clerkship. The Human Subjects Committee of Children's National Hospital approved this study as expedited.

Statistical methods first addressed the comparability of the 2 study groups. Methods used to evaluate evidence of baseline difference across study groups depended on the nature and distribution of the characteristic being compared. Categorical comparisons were implemented by chi square tests, and comparisons based on measurements by analysis of variance or nonparametric procedures for non-normally distributed data. Analyses of outcomes were based on linear regression analysis using bootstrapping methods, with 1,000 replications, to generate model parameter and variance estimates which do not depend on the normality assumption. The study was powered at 80% to detect a moderate effect size (0.5 SD) difference in scored results between 2 randomly assigned groups with a 2-tailed type 1 error of 5%. A sample size of at least 64 per group was planned to address study aims.

## Results

**Figure 1:**
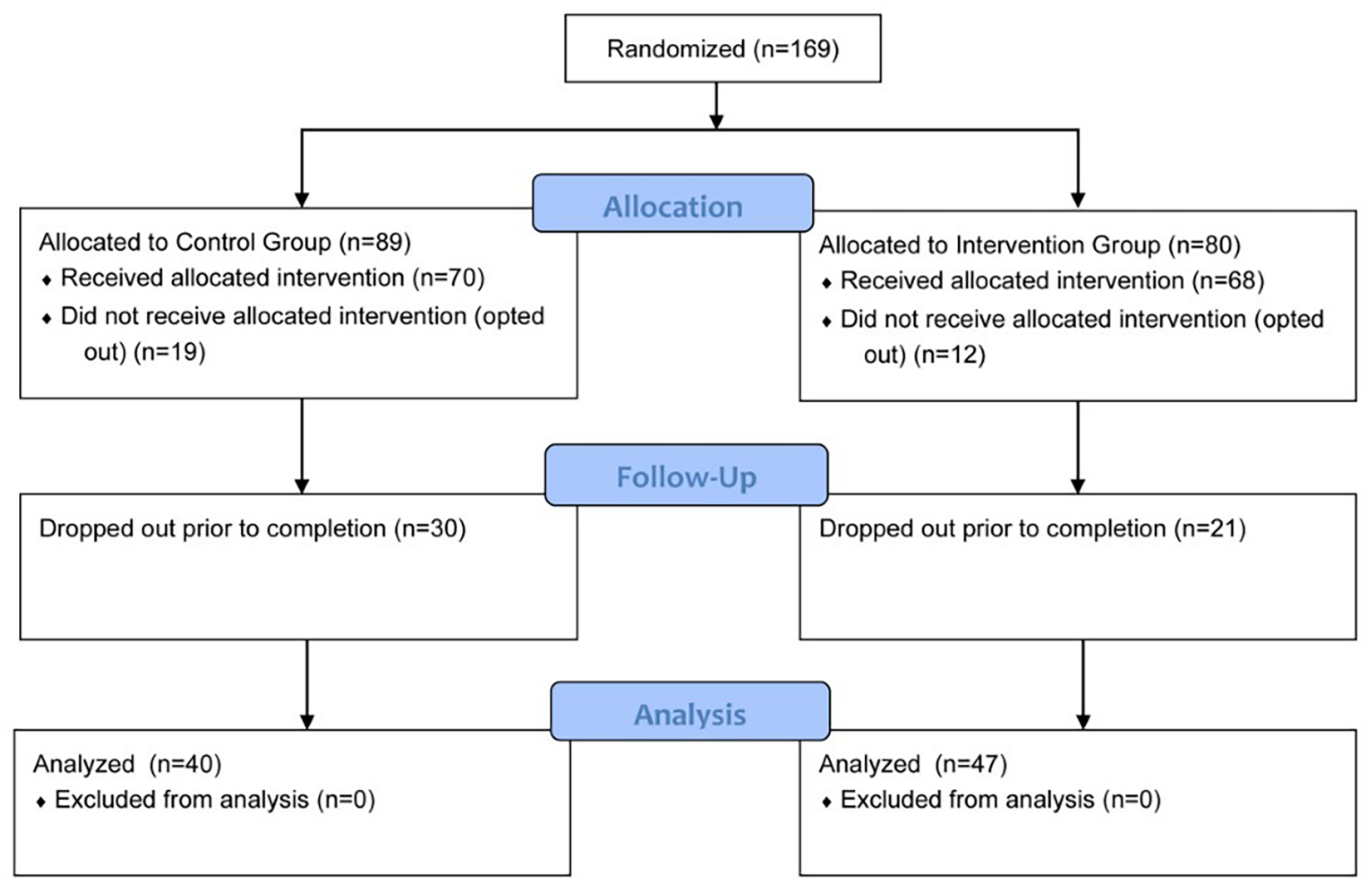
Subject enrollment.

All 3rd-year GWU medical students participating in their pediatric clerkship over a 10-month period were randomized 1:1 to the Control group (regular training) or the Intervention group (regular training + modules). Of these, 138 subjects took the pre-test; 87 also completed the post-test, yielding interpretable data regarding improvement. Of those completing both tests, 47 had been randomized to Intervention and 40 to Control (Figure 1).

Table 1 compares full participants and drop-outs by group on demographics, prior experience, self-assessment and pre-test performance. Full participants in the intervention and control groups were very similar in all comparisons.

**Table 1:** Sample characteristics.

Variable	Intervention group			Control group			Intervention control
	Full participants (*N* = 47) *N* (%)	Drop-outs (*N* = 21) *N* (%)	Full v. Drop-out *p*-value	Full participants (*N* = 40) *N* (%)	Drop-outs (*N* = 30) *N* (%)	Full v. Drop-out *p*-value	
Age (years)			0.14^a^			0.34^a^	0.60^a^
20–24	21 (44.7)	5 (26.3)		14 (35.0)	6 (20.0)		
25–29	23 (48.9)	10 (52.6)		22 (55.0)	19 (63.3)		
30–39	3 (6.4)	4 (21.1)		4 (10.0)	5 (16.7)		
Gender			0.72^a^			0.94^a^	0.90^a^
Female	30 (63.8)	11 (57.9)		25 (62.5)	19 (63.3)		
Male	17 (36.2)	8 (42.1)		15 (37.5)	11 (36.7)		
Previous experience in learning auscultation			0.64^a^			0.53^a^	0.78^a^
No exposure	6 (12.8)	3 (14.3)		5 (12.5)	2 (6.7)		
Multimedia	30 (63.8)	11 (52.4)		28 (70.0)	20 (66.7)		
Other exposure	11 (23.4)	7 (33.3)		7 (17.5)	8 (26.7)		
“Skill at cardiac auscultation is important to my future”			1.00^b^			1.00^b^	0.50^b^
Agree or strongly agree	45 (95.7)	19 (100.0)		40 (100.0)	30 (100.0)		
Disagree, strongly disagree	2 (4.3)	0 (0.0)		0 (0.0)	0 (0.0)		
“My current skills in cardiac auscultation are good”			0.58^b^			0.45^b^	0.66^b^
Agree	2 (4.3)	2 (10.5)		3 (7.5)	4 (13.3)		
Disagree, strongly disagree	45 (95.7)	17 (89.5)		37 (92.5)	26 (86.7)		
Hearing problem			1.00^b^			0.57^b^	1.00^b^
Yes	2 (4.3)	0 (0.0)		1 (2.5)	2 (6.7)		
No	45 (95.7)	21 (100.0)		39 (97.5)	28 (93.3)		
Pre-test results							
CDIP (%), median (IQR)	10 (0, 26)	10 (0, 21)	1.00^c^	12.5 (0, 25)	10 (0, 29)	0.98^c^	0.98^c^
DIP (%), mean (sd)	59.7 (14.0)	52.9 (10.9)	0.05^d^	61.3 (10.9)	59.0 (10.6)	0.88^d^	0.55^d^
Specific diagnosis (%), mean (sd)	21.9 (15.4)	14.3 (11.1)	0.05^d^	22.4 (13.5)	21.4 (17.6)	0.79^d^	0.87^d^

CDIP, distinguishing innocent from pathological with at least moderate confidence; DIP, distinguishing innocent from pathological; IQR, Interquartile range. SD: Standard deviation.

^a^ Pearson chi-square test.

^b^ Fisher's exact test.

^c^ Median test.

^d^ *t*-test.

**Figure 2:**
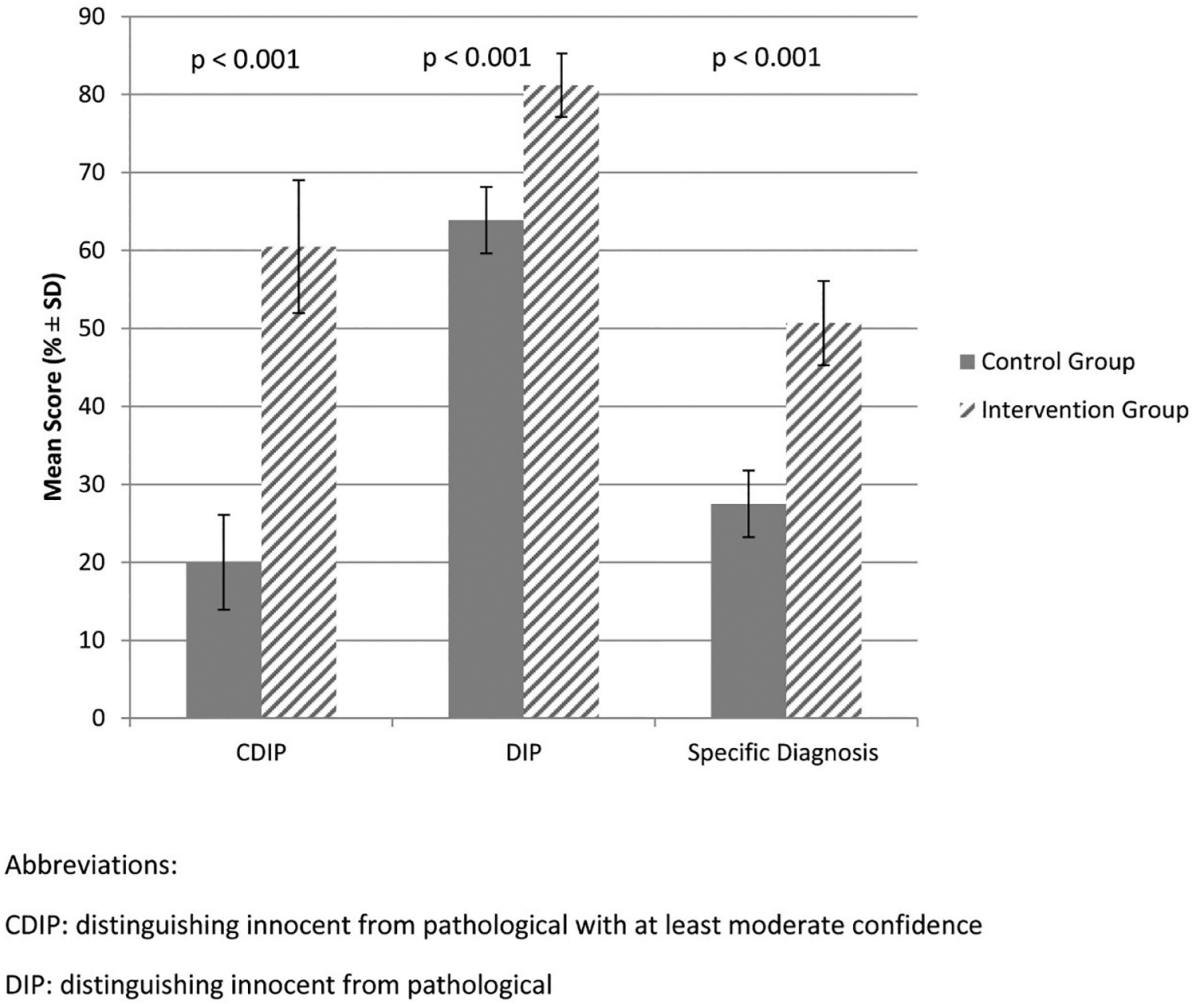
Effect of intervention on 3 study outcomes.

Figure 2 compares post-test scores (95% confidence interval) by group, controlling for pre-test scores. In the primary outcome (CDIP), the Intervention group (I) received a score of 60.5% (51.8, 69.1) compared to the Control (C) group's 20.0% (14.1, 26.0) (*p* < 0.001). The comparable results for the two secondary outcomes, DIP (I group 81.2% (77.1, 85.3) vs. C group 63.9% (59.6, 68.2) (*p* < 0.001)) and specific diagnosis I group 50.7% (45.1, 56.2) vs. C group 27.5% (23.3, 31.7, *p* < 0.001), were also significantly different. Controlling for pre-test confidence scores, post-test confidence scores (range 20–80) were significantly higher in the Intervention group 57.7 (53.1, 62.2), compared to the Control group 41.4 (37.7, 45.1) (*p* < 0.001).

**Figure 3:**
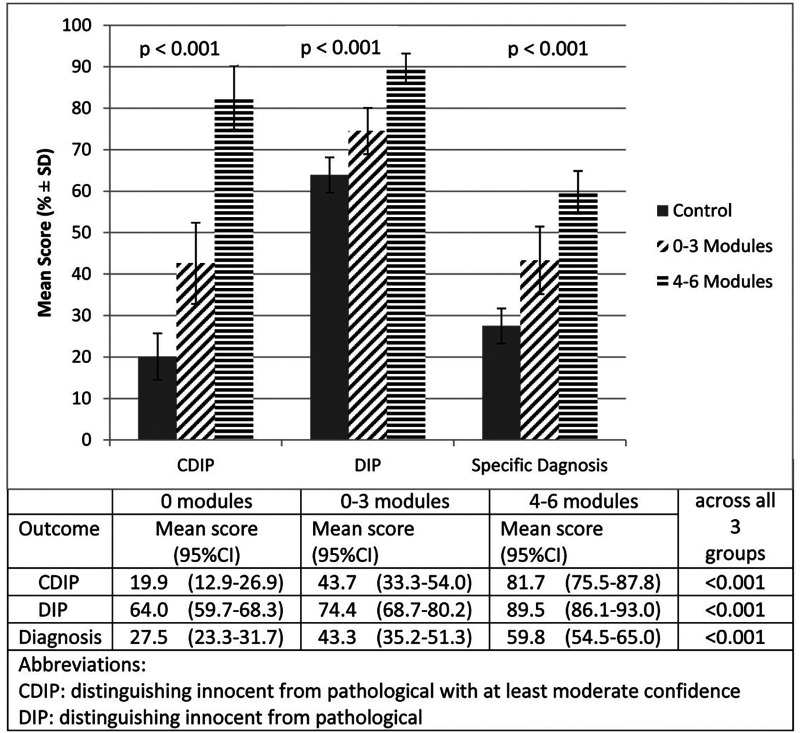
Effect of intervention by number of modules accessed.

Of the 47 participants in the Intervention group, 3 subjects did not access any modules. Twenty-six accessed more than the required 2 modules, and of these, 20 accessed all 6. Controlling for pre-test scores, those subjects who accessed more modules scored significantly higher on the post-test for all outcomes than those who did fewer, and those who accessed 0–3 modules scored significantly higher than those randomized to the Control group (Figure 3).

Satisfaction feedback was obtained anonymously from the subjects at the end of the post-test. Due to malfunction of the data-collection system, the Likert responses were not recorded. However, detailed responses were obtained from all subjects to the following questions: “Do you feel that your skill in evaluating heart murmurs has improved over the course of your clerkship?” and “Which if any of the following did you use to supplement your auscultatory learning during the clerkship?” The categorical results of the former are given in Table 2, demonstrating a strongly significant effect of the intervention (“Yes” in 70% of the intervention group v. 15% of controls). Sample comments are listed in Supplementary Table S3.

**Table 2:** Feedback regarding improvement.

Group	“Do you feel that your skill in evaluating heart murmurs has improved over the course of your clerkship?”				*p*-value
	Yes	No	Maybe	n/a	
Control *N *= 40	6 (15%)	28 (70%)	6 (15%)	0	<0.001
Intervention *N* = 47	33 (70%)	4 (9%)	6 (13%)	4 (9%)	<0.001

Members of the Control group often responded to the effect, “I haven't had access to the modules yet”. Only one control subject reported improvement over the 4 weeks between the tests, attributed to a cardiology lecture given to all the students.

Responses from the Intervention group were largely very positive Four members of this group reported that they did not think they improved significantly, and two others that they did not have the time to do the modules.

There were no significant differences between the 2 groups in use of supplementary learning materials (Supplementary Table S2).

## Discussion

This study demonstrates the substantial impact of a new teaching method on the skill of medical students in differentiating innocent murmurs from pathological murmurs in children. Subjects viewing even 1 or 2 modules performed significantly better on all 3 outcomes measured than the control group. Moreover, those subjects who viewed more modules showed progressively better acquisition of this skill.

We believe this is the first pediatric CA program evaluated with a randomized controlled trial comparing pre- and post-intervention scores on standardized tests. Finley et al. (28) designed a 1-hour audio-only training program for 124 medical students in Australia and Nova Scotia. Utilizing a complex 7-level interactive self-paced approach and asking for a binary innocent/pathological choice, they demonstrated a small but significant improvement from 75% (Australian) and 85% (Canadian) to 95% on the post-test. In choosing our primary outcome, we avoided such high baseline scores, which likely reflected educated guesses. Murmur files for both their training program and their tests were chosen randomly from one pool of 56 recordings; no 2 subjects and no 2 tests were the same, rendering comparisons problematic. Germanakis et al. (11) tested 106 primary care practitioners before and after an 8-hour intensive seminar including lectures and multimedia. The subjects showed substantial improvement in the binary choice, except in the recognition of innocent murmurs, rising from a baseline of 17% to only 26%. Their study was not randomized and had no control group.

We selected CDIP as our primary outcome as a surrogate for differentiating between those patients who would be referred to a cardiologist and those who would not. Thus, “correct” answers in which the subjects had little or no confidence were counted as incorrect, because these murmurs would have prompted a referral. The ability to make a specific auscultatory diagnosis (e.g., aortic stenosis), while significantly better in the Intervention group, is of less importance in the clinical setting, where the PCP must make a simple binary decision (31, 32). The emphasis on this binary decision has been embraced by several investigators studying pediatric CA (11, 15, 17, 24, 25) and adult cases (42).

Other pediatric murmur programs targeting students and trainees (16, 26, 27) have focused on the more challenging tasks of identifying auditory features (such as shape or quality) and/or reaching an actual diagnosis, which in practice is usually subsequently reached by a pediatric cardiologist with or without other data. We chose not to score our learners on features of the murmurs, as has been done in other such tests (18, 26, 43), in which identification of such features correlates poorly with recognition of the innocence, or the cause, of the murmur (22, 27). We believe that identification of these details does not aid the learner in recognizing murmurs, but adds another layer of cognitive material to master which is not essential to the purpose.

The effect size demonstrated here was clinically significant, with the Intervention subjects scoring 60.5% correct on the primary outcome, compared with 20.0% in the Control group. This is in contrast with published studies of pediatric murmur programs (11, 26) in which the improvement in this binary distinction was statistically significant but was not substantive relative to the investment in time (6 and 8 h, respectively), personnel, and funding.

We did not propose an arbitrary target score to define mastery. However, we noted that the post-test scores for the Intervention group on all outcomes fell between those of 11 cardiology faculty and fellows and those of 6 non-cardiology pediatric faculty members (mean scores for primary outcome 90.0% and 55.0%, respectively) studied in the pilot project. Furthermore, those subjects who accessed 4–6 modules performed similarly to the cardiologists with respect to our primary outcome. This result exceeded our expectations.

The pre- and post-tests evaluated the *external* validity of this skill, by providing “new” recordings which were not used in the teaching modules. In other studies (14, 28, 44) investigators have used the same recordings for both training and testing purposes, possibly testing the subject's ability to recognize the actual recording, which reflects only internal validity.

For the teaching modules, we used multiple recordings to illustrate each common murmur, in contrast to the work of Barrett et al. (14), in which precise repetition of a single cardiac cycle is deliberately employed, with the goal of forming an auditory template for the learner. To enable our learners to more accurately categorize a “new” murmur when it is encountered, we chose to train them with a range of different recordings of each murmur (33).

We approximated the clinical setting by providing more than one “case” of each common diagnosis on the tests, so that subjects could not use one answer to deduce another by elimination, which might allow learners to game the system. Similarly, we offered the same 8 options for diagnoses of pathological murmurs in all test questions, making a correct guess less likely than with the standard 4 or 5 options which vary between cases (45).

### Limitations

Because our subjects were randomized prior to self-enrollment by completing the pre-test, we cannot determine whether those who opted out (18% of the total randomized group) were comparable to the population who participated. However, we found no significant differences between those who initiated but did not complete participation, and those who did complete the study (Table 1).

The quantitative effect of increasing exposure to the modules is an observation based on non-randomized self-selection of students within the Intervention group, and therefore subject to selection bias.

We did not assess for retention of this skill by re-testing the subjects after a time interval. This curriculum, being brief and readily accessible, was designed to be made available for reinforcement learning after the initial exposure (28).

Transferability to the clinical setting was not tested in this study, nor in any other published studies of teaching CA. Our choice of 3rd-year students as subjects was guided by a desire to have an early impact on the learning of this clinical skill, and did not permit that type of assessment.

Our data regarding learner satisfaction was limited, but largely positive. The fact that over half of the Intervention subjects chose to do more than the 2 “required” modules, and 43% of the group completed all 6 modules, demonstrates change in behavior and attests to its value to them.

We elected for simplicity (Supplementary Figures S1–S5) in designing the program, to optimize multisensory linkage while minimizing cognitive load, given the goal to provide opportunity for deliberate practice as a means of learning auditory recognition, not pathophysiology (46). This less cluttered approach also helps to avoid the intimidation which may be experienced by users of more complex teaching programs. However, we cannot determine which of the features of our program contributed most to the outcome.

### Future directions

We plan to test this program on a different, more advanced group of learners, with late follow-up to evaluate both retention and the booster effect of the modules. At present, it is incorporated into the pediatric clerkship for 3rd-year medical students at GWU, and into the pediatric cardiology rotation of 2nd-year pediatric residents from the Children's National and Georgetown University programs. Further, we hope to disseminate it more widely to pediatric trainees and practitioners in the future.

## Summary

We have created a novel teaching program using actual recordings of pediatric murmurs to aid medical trainees in learning to recognize common murmurs in children, incorporating major principles of andragogy and multimedia learning in an effort to avoid the pitfalls of previous such innovations and their assessments (19). We demonstrated its effectiveness in a randomized controlled trial to result in a meaningful gain in this skill from 1 h of self-paced individualized training with high acceptance to learners.

## Data Availability

The raw data supporting the conclusions of this article will be made available by the authors, without undue reservation.
