# Supplementary material for: A randomized, controlled trial of an innovative, multimedia instructional program for acquiring auditory skill in identifying pediatric heart murmurs
Source: Front Pediatr. 2024 Jan 16;11:1283306. doi: 10.3389/fped.2023.1283306 (PMC10825047; doi:10.3389/fped.2023.1283306)
Supplement: Supplementary file 1 [file Table1.docx]

Table S1 Titles of learning modules

1. Still’s Murmur
2. Ventricular Septal Defects
3. Venous Hum
4. Patent Ductus Arteriosus
5. Obstructive Lesions
6. Valvar Insufficiency
