# Supplementary material for: A randomized, controlled trial of an innovative, multimedia instructional program for acquiring auditory skill in identifying pediatric heart murmurs
Source: Front Pediatr. 2024 Jan 16;11:1283306. doi: 10.3389/fped.2023.1283306 (PMC10825047; doi:10.3389/fped.2023.1283306)
Supplement: Supplementary file 2 [file Table2.docx]

Table S2. Use of supplementary learning materials by subjects

| “Which if any of the following did you use to supplement your  auscultatory learning during the rotation?” | | | |
| --- | --- | --- | --- |
| SUPPLEMENTARY SOURCES USED | CONTROL | INTERVENTION | TOTAL |
| Bedside teaching | 23 (58%) | 24 (51%) | 47 (54%) |
| Formal lectures | 8 (20%) | 13 (28%) | 21 (24%) |
| Audio recordings | 4 (10%) | 8 (17%) | 12 (14%) |
| On-line or CD-ROM program | 3 (8%) | 0 | 3 (3%) |
| Simulations | 0 | 0 | 0 |
| None | 13 (33%) | 17 (36%) | 30 (34%) |
| Control v. Intervention, p-value = NS | | | |
