# Supplementary material for: A randomized, controlled trial of an innovative, multimedia instructional program for acquiring auditory skill in identifying pediatric heart murmurs
Source: Front Pediatr. 2024 Jan 16;11:1283306. doi: 10.3389/fped.2023.1283306 (PMC10825047; doi:10.3389/fped.2023.1283306)
Supplement: Supplementary file 3 [file Table3.docx]

Table S3 Feedback regarding subject satisfaction

| **Study Group** | **Comments** |
| --- | --- |
| Control | “I think if I had been in the experimental group I would have but it is very hard to get better at hearing murmurs unless you practice on your own with programs like this one.” |
| Control | Only one control subject reported improvement over the 4 weeks of clerkship between the tests, attributing that to a cardiology lecture given to all the students. |
| Intervention | “This…was the best practical training we have received in screening for and evaluating heart murmurs.” |
| Intervention | “This module was super helpful! I would probably recommend this to everyone.” |
| Intervention | “The repetition built into the modules is key.” |
| Intervention | “I haven’t heard so many heart sounds in one place before…the pictures were all simply presented and very helpful.” |
| Intervention | “The comparisons to actual sounds (groans, faucet running) were very helpful. Loved these learning modules!” |
| Intervention | Four intervention subjects simply reported that they did not think they improved significantly, and two others that they did not have the time to do the modules. |
