# Supplementary figures and images for: A randomized, controlled trial of an innovative, multimedia instructional program for acquiring auditory skill in identifying pediatric heart murmurs
Source: Front Pediatr. 2024 Jan 16;11:1283306. doi: 10.3389/fped.2023.1283306 (PMC10825047; doi:10.3389/fped.2023.1283306)

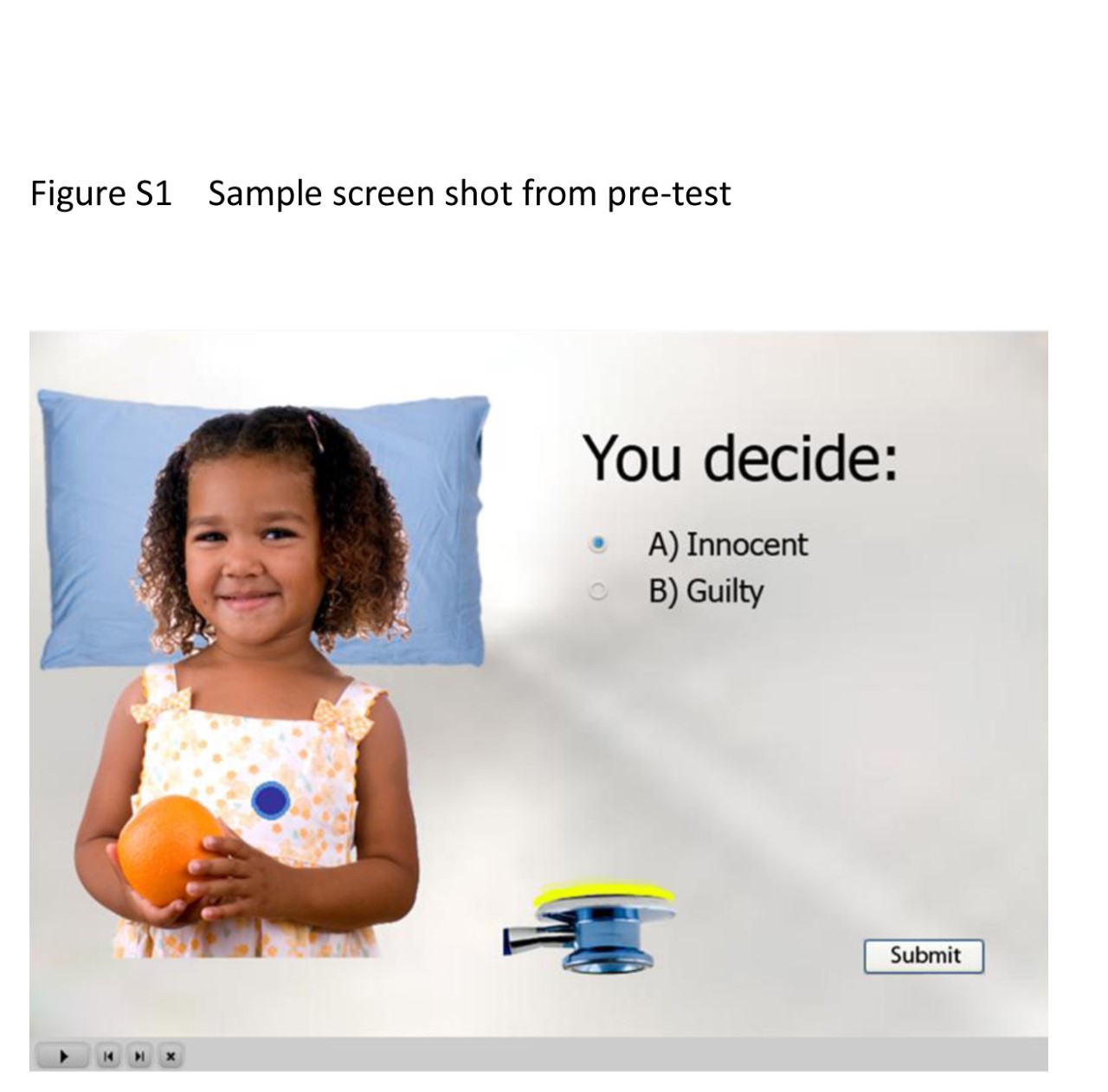

Supplement: Supplementary file 4 [file Image1.jpeg]

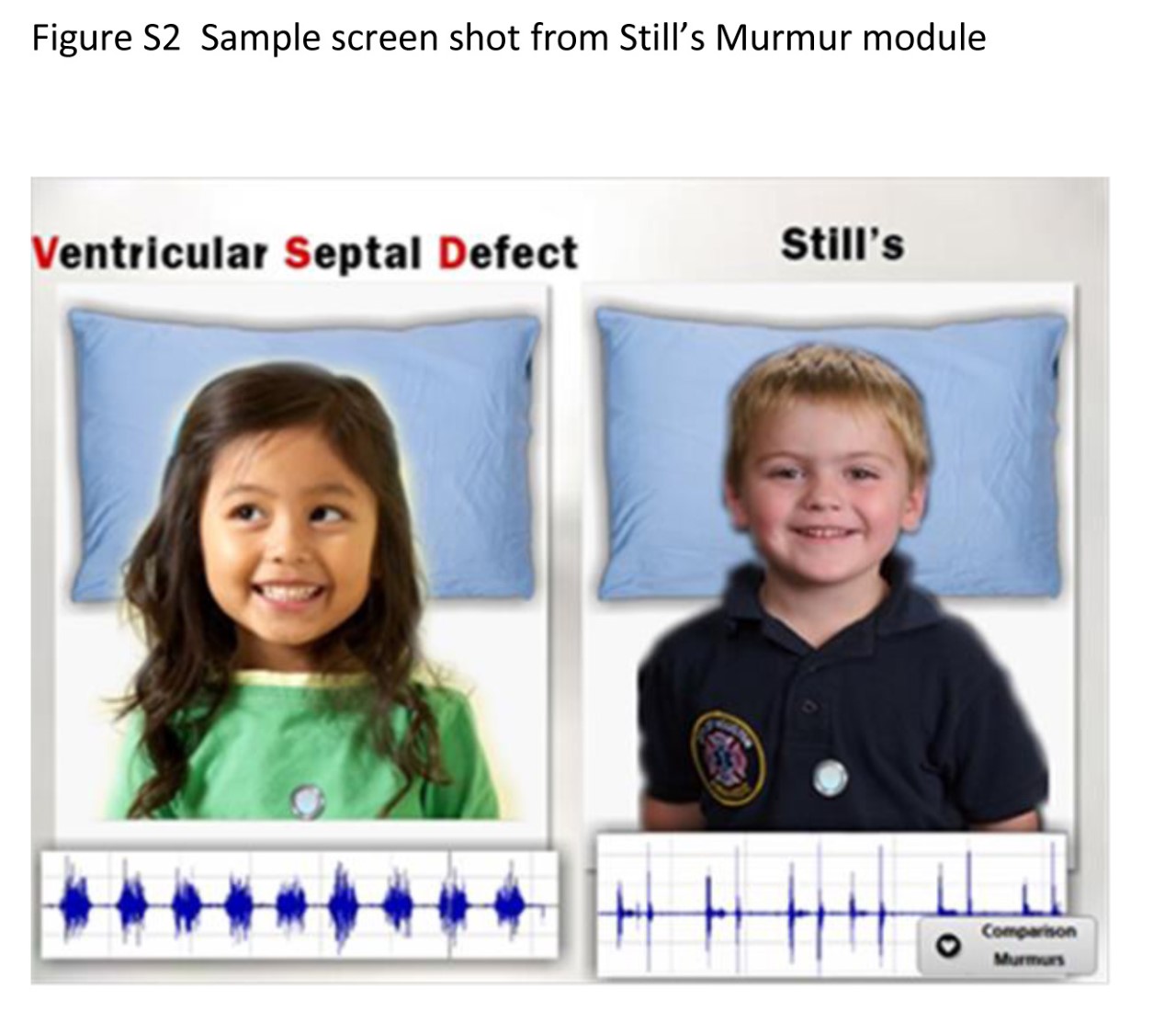

Supplement: Supplementary file 5 [file Image2.jpeg]

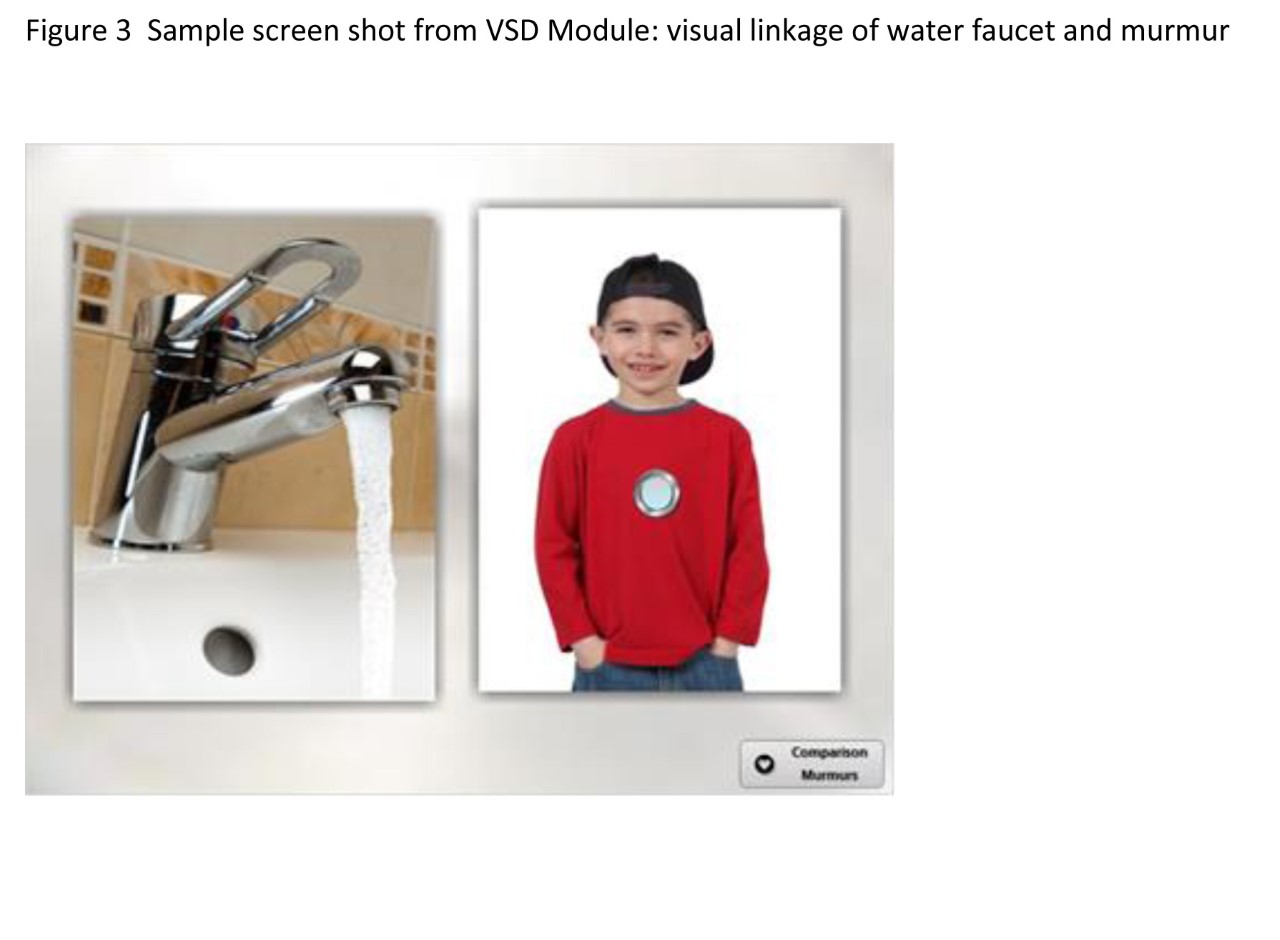

Supplement: Supplementary file 6 [file Image3.jpeg]

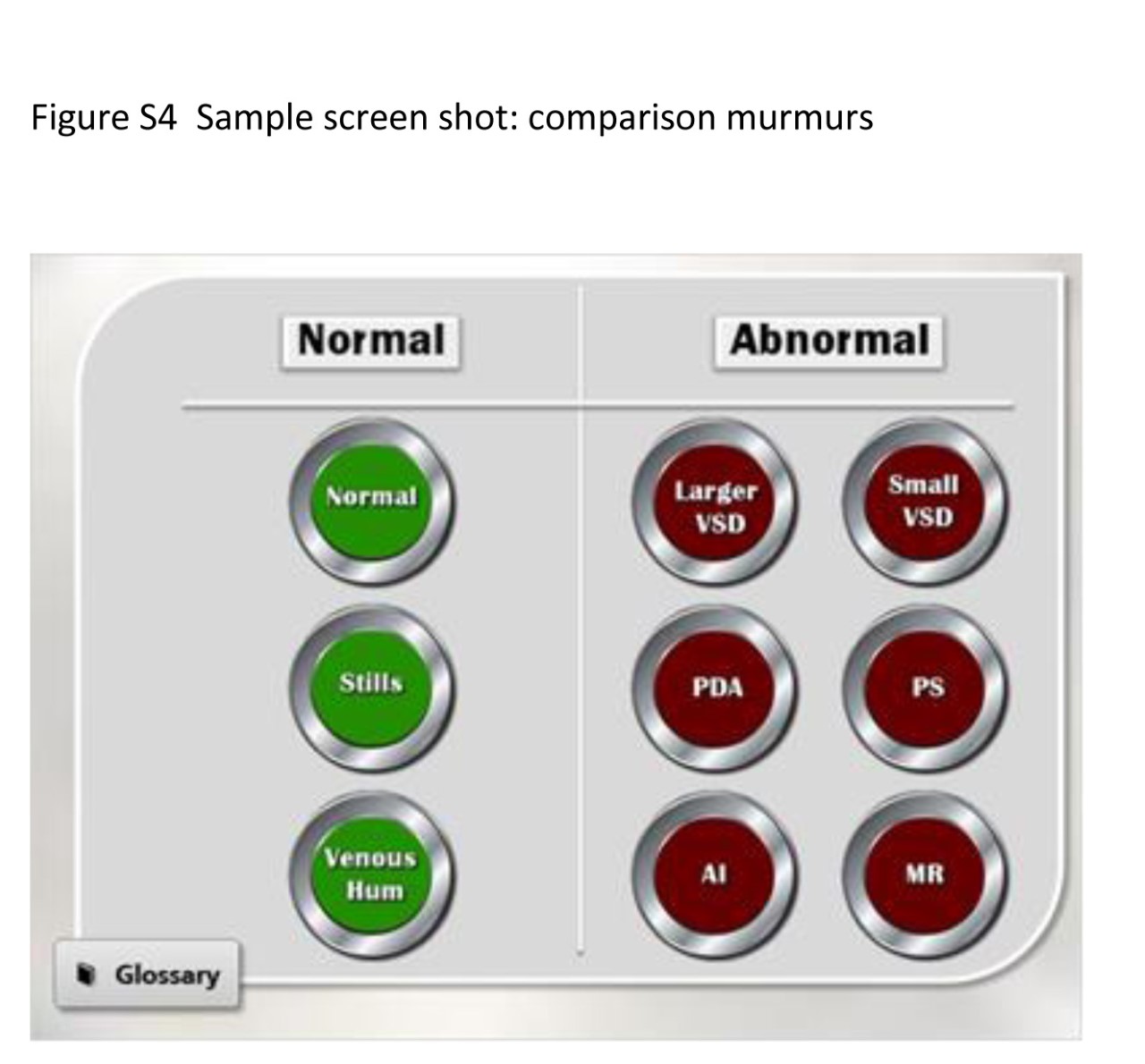

Supplement: Supplementary file 7 [file Image4.jpeg]

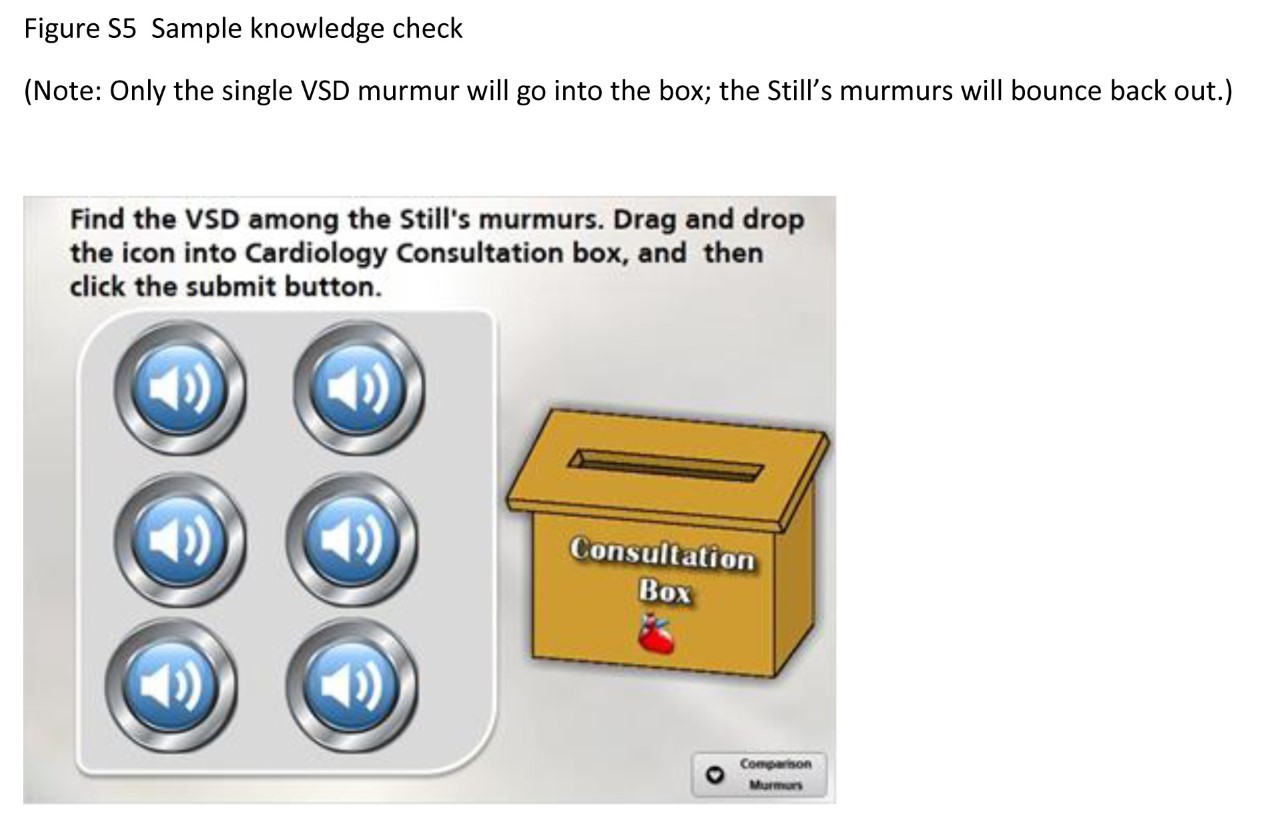

Supplement: Supplementary file 8 [file Image5.jpeg]
